# Increased Tim-3 expression on peripheral T lymphocyte subsets and association with higher disease activity in systemic lupus erythematosus

**DOI:** 10.1186/s13000-015-0306-0

**Published:** 2015-06-16

**Authors:** Li-jun Song, Xiao Wang, Xu-ping Wang, Dong Li, Feng Ding, Hua-xiang Liu, Xiao Yu, Xing-fu Li, Qiang Shu

**Affiliations:** Department of Rheumatology, Qilu Hospital of Shandong University, 107 Wenhua Xi Road, Ji’nan, 250012 China; The Key Laboratory of Cardiovascular Remodeling and Function Research, Chinese Ministry of Education and Chinese Ministry of Health, Qilu Hospital of Shandong University, Ji’nan, 250012 China; Cryomedicine Laboratory, Qilu Hospital of Shandong University, Ji’nan, 250012 China

**Keywords:** Systemic lupus erythematosus, Tim-3, Fas, T lymphocyte subset, Disease activity

## Abstract

**Background:**

Both the T cell immunoglobulin domain- and mucin domain-containing molecule-3 (Tim-3) and the death receptor Fas contribute to the pathogenesis of various autoimmune diseases, including systemic lupus erythematosus (SLE). The aim of the present study was to determine whether Tim-3 and Fas are co-expressed on certain peripheral T lymphocyte subsets, and whether this expression is associated with greater disease activity in SLE.

**Methods:**

Peripheral blood mononuclear cells were isolated from 46 patients newly diagnosed with SLE and 28 age- and sex-matched healthy controls (HCs). Expression of Tim-3 and Fas on T subsets was analyzed by flow cytometry, while mRNA levels of the Tim-3 ligand galectin-9 and Fas ligand FasL were assayed using real-time RT-PCR.

**Results:**

The proportions of CD3^+^CD4^+^ and CD3^+^CD4^-^ T cells expressing Tim-3^+^ and Tim^+^Fas^+^ were significantly higher in patients than in HCs (p < 0.05), while the proportions of these subtypes expressing Fas were similar for the two groups. Patients with active SLE, as defined by their score on the SLE Disease Activity Index, had lower proportions of CD3^+^CD4^+^ T cells and higher proportions of CD3^+^CD4^+^Tim-3^+^ and CD3^+^CD4^+^Tim-3^+^Fas^+^ T cells than did patients with stable SLE. Serum levels of complement C3 and C4 proteins, considered as a marker of SLE activity, correlated negatively with proportions of CD3^+^CD4^+^ and CD3^+^CD4^-^ T cells expressing Tim-3.

**Conclusions:**

Expression of Tim-3 and co-expression of Tim-3 and Fas on certain peripheral T subsets are associated with disease activity in SLE patients. Future research should examine whether the same is true of other T subsets implicated in SLE, and should explore the potential role(s) of Tim-3 in the disease pathway.

**Virtual slides:**

http://www.diagnosticpathology.diagnomx.eu/vs/1855527845145188

## Background

Systemic lupus erythematosus (SLE) has been extensively studied as a disease of adaptive immunity. T cell dysfunction, cytokine production [1–4] and dysregulation of cell apoptosis [5, 6] are known to play pivotal roles in SLE, but how the disease begins and progresses is unclear.

Signaling mediated by the Fas receptor and its natural ligand, FasL, contribute to the pathogenesis of SLE [5–8]. Such signaling contributes to the pathogenesis of various autoimmune diseases by promoting apoptosis and immune escape [9]. Fas promotes the removal of activated T cells via activation-induced cell death, while FasL, a member of the tumor necrosis factor super family, initiates the death signaling cascade and promotes apoptosis [10]. Peripheral blood T lymphocytes show higher apoptosis rates in patients with SLE than healthy controls (HCs), and this correlates with greater mRNA levels of Fas [11, 12] and FasL [13, 14].

T cell immunoglobulin domain- and mucin domain-containing molecule-3 (Tim-3), a member of the Tims family, has been implicated in the pathogenesis of asthma, type 1 diabetes, collagen-induced arthritis and other autoimmune diseases [15–19]. Whether it also participates in SLE is unclear. Tim-3 is expressed primarily on the surface of activated Th1 cells, and it binds to its ligand galectin-9 to negatively regulate interferon-γ (IFN-γ) secretion [20, 21]. Other immune cells can also express Tim-3,such as cytotoxic T lymphocytes (Tcl), Th17, macrophages, dendritic cells and natural killer T cells; such expression affects immune functions in mice and humans [22, 23]. Tim-3 functions as the phagocytic receptor to remove apoptotic cells [24], while binding of galectin-9 to the carbohydrate chains of Tim-3 has been shown to induce Th1 cell death in vitro [21].

Previous work from our laboratory showed that peripheral blood mononuclear cells (PBMCs) from patients with SLE express similar levels of Tim-3 mRNA as PBMCs from HCs [25]. However, whether Tim-3 expression is similar across the various subsets of PBMCs, including T lymphocytes, B lymphocytes and monocytes, is unclear. In addition, since both Tim-3 and Fas are apoptosis-associated surface molecules, it would be important to investigate whether the expression of one correlates with expression of the other on peripheral T subsets in patients with SLE relative to HCs. Such studies may help provide insights into SLE pathogenesis.

Therefore, in the present study, we quantified the proportions of the two main peripheral T subsets cells expressing Fas and/or Tim-3 in patients with SLE and HCs. We also examined mRNA expression of the corresponding receptor ligands galectin-9 and FasL in PBMCs. Finally, we examined possible relationships of Tim-3/Galectin-9 and Fas/FasL expression with SLE disease activity.

## Methods

### Patients and HCs

A total of 46 patients newly diagnosed with SLE according to revised criteria from the American College of Rheumatology for the classification of systemic lupus erythematosus [26] were included in the study. Patients were admitted to our hospital from June 2009 to March 2010. Clinical disease activity was assessed at the time of measurement according to the systemic lupus erythematosus disease activity index (SLEDAI) 2000. Patients with a SLEDAI score ≥ 10 were defined as having active SLE [27], while patients with lower SLEDAI scores were defined as having stable disease. As a reference group, 28 age- and sex-matched HCs were enrolled. The study was approved by the Institutional Review Board of Qilu Hospital of Shandong University. All patients and HCs gave informed consent for participation in the study.

### Flow cytometry

Fresh PBMCs were collected from patients with SLE and HCs and prepared for fluorescence-activated cell sorting (FACS). The cell surface was stained using the following fluorochrome-conjugated monoclonal antibodies: anti-CD3 PE-Cy5, anti-CD4 FITC, anti-Fas-APC (mouse IgG1, BD Biosciences, USA) and anti-Tim-3 PE (rat IgG2A, R&D, USA). Cells were then fixed and permeabilized with RBC lysis buffer (BD Biosciences). Gates and quadrants were set based on isotype control staining (BD Biosciences and R&D). Data were acquired using multicolor analysis on the FACS Calibur flow cytometer (BD Biosciences) and analyzed by FlowJo software 7.6.2 (Tree Star, Ashland, OR, USA).

### Real-time quantitative reverse transcription-PCR to measure galectin-9 and FasL mRNA levels

PBMCs were isolated from peripheral blood using density gradient centrifugation based on Ficoll (Haoyang Biosciences, Tianjin, China). Total cellular RNA was extracted using Trizol reagent (TransGen Biosciences, Beijing, China). Reverse transcription was performed using the TransScript First-Strand cDNA Synthesis SuperMix kit (TransGen Biosciences). Then amplification was performed using an AB 7500 Real-Time PCR System (AB Biosciences, CA, USA) following the manufacturer’s instructions. The Power SYBR Green PCR Master Mix kit (AB Biosciences) was used with the following two-step PCR protocol: 95 °C for 10 min, followed by 40 cycles of 95 °C for 15s and 60 °C for 1 min.

The following primers were used for galectin-9: forward, 5′-ACTTTCAGAACAGCTTCAATGGA-3′; reverse, 5′-AGTCCATCATGATATCAGGCAAT-3′. The following primers were used for FasL: forward, 5′-CAATGAAAATGAACACATTG-3′; reverse, 5′CCCACTTTAGAAATTAGATC-3′. As an internal control, β-actin mRNA was amplified using the following primers: forward, 5′-TTGCCGACAGGATGCAGAA-3′; reverse, 5′-GCCGATCCACACGGAGTACT-3′. Analysis of PCR products on 5 % agarose showed the expected sizes. All reactions were run in parallel in the absence of reverse transcriptase to exclude the possibility of genomic contamination.

### Serology

Serum levels of complement 3 (C3) and serum complement 4 (C4), the presence of dsDNA and anti-nuclear antibodies (ANA), and the total number of white blood cells (WBCs) were assayed in the clinical laboratory of Qilu Hospital. SLEDAI scores were recorded at the time of follow-up for SLE patients.

### Statistical analysis

All values are expressed as mean ± SD unless stated otherwise. Inter-group differences were assessed for significance using the Mann-Whitney *U*-test. Spearman’s rank correlation was used to examine possible correlations between patient variables and presence or activity of SLE. The threshold for significance was defined as *p* < 0.05. All statistical analyses were performed using GraphPad Prism 5.00.

## Results

### Demographic and clinical characteristics in patients with SLE and HCs

Patients and HCs showed no significant differences in age or sex distribution (Table 1). All patients were positive for ANA, whereas all HCs were negative; hypocomplementemia was present in 65.2 % of patients. The average number of total WBCs in circulation was lower in patients than in HCs (5.15 ± 2.91 *vs.* 5.88 ± 1.34 cells/nl), though the difference did not achieve significance (*p* = 0.152). The proportion of lymphocytes in WBCs was significantly lower in patients (15.54 ± 10.93 *vs.* 21.26 ± 5.86, *p* = 0.005).

**Table 1:** Clinical characteristics and proportions of lymphocyte subsets in peripheral blood of Chinese patients with SLE and healthy controls

	SLE	Controls
N	46	28
Age, yr	34.6 ± 12.7	34.8 ± 13.6
Female, n (%)	42 (91.3)	25 (89.3)
Male, n (%)	4 (8.7)	3 (10.7)
ANA (+), n (%)	46 (100)	0
C_3_, g/L	0.76 ± 0.36	-
C_4_, g/L	0.16 ± 0.12	-
SLEDAI score	8.6 ± 4.3	-
WBCs (×10^9^/L)	5.15 ± 2.91	5.88 ± 1.34
Lymphocytes, %^a^	15.54 ± 10.93^*^	21.26 ± 5.86
CD3^+^CD4^+^ T subset, %^b^	21.96 ± 9.11^*^	30.58 ± 7.75
CD3^+^CD4^-^ T subset, %^b^	26.28 ± 11.81	24.27 ± 7.99
Ratio of CD3^+^CD4^+^/CD3^+^CD4^-^ T	0.97 ± 0.50^*^	1.38 ± 0.54

Values shown are mean ± SD, unless otherwise noted

*ANA*, anti-nuclear antibody; *C*, complement; *SLEDAI*, systemic lupus erythematosus disease activity index; *WBCs*, white blood cells

^a^Percentage of total white blood cells in peripheral blood

^b^Percentage of total lymphocytes in peripheral blood

*p < 0.05 compared to healthy control group

Among patients, 21 (45.6 %) were classified as having active SLE based on an SLEDAI score ≥ 10. Dividing the patients into groups with active or stable disease revealed no significant differences in age or sex distribution (Table 2). The two groups did differ significantly in SLEDAI score, incidence of dsDNA positivity and serum levels of C3 and C4 (*p* = 0.005).

**Table 2:** Demographic and clinical data for Chinese patients with active or stable SLE

	Active SLE	Stable SLE
N	21	25
Age, yr	33.5 ± 14.2**	36.2 ± 12.3
Female, n (%)	19 (90.5)**	23 (92.0)
Male, n (%)	2 (9.5)**	2 (8.0)
ANA (+), n (%)	21 (100)	25 (100)
dsDNA (+), n (%)	14(67)*	6(24)
C_3_, g/L	0.64 ± 0.41*	0.94 ± 0.35
C_4_, g/L	0.13 ± 0.11**	0.20 ± 0.13
SLEDAI score	13.9 ± 3.2*	5.4 ± 2.1

Values shown are mean ± SD unless otherwise noted

*ANA*, anti-nuclear antibody; *C*, complement; *SLEDAI*, systemic lupus erythematosus disease activity index

*p < 0.05 compared to stable SLE group

**p > 0.05 compared to stable SLE group

### CD3^+^CD4^+^T lymphocyte subsets in patients with SLE and HCs

Patients showed a significantly lower proportion of circulating CD3^+^CD4^+^ T cells than HCs did (21.96 ± 9.11*vs.*30.58 ± 7.75, *p* < 0.001; Table 1), where as the proportion of CD3^+^CD4^-^ T cells was similar between the two groups. As a result, the ratio of CD3^+^CD4^+^ cells to CD3^+^CD4^-^ T cells was significantly lower in patients (0.97 ± 0.50 *vs.* 1.38 ± 0.54, *p* = 0.002).

### Tim-3 expression on T cell subsets in patients and HCs

**Fig. 1:**
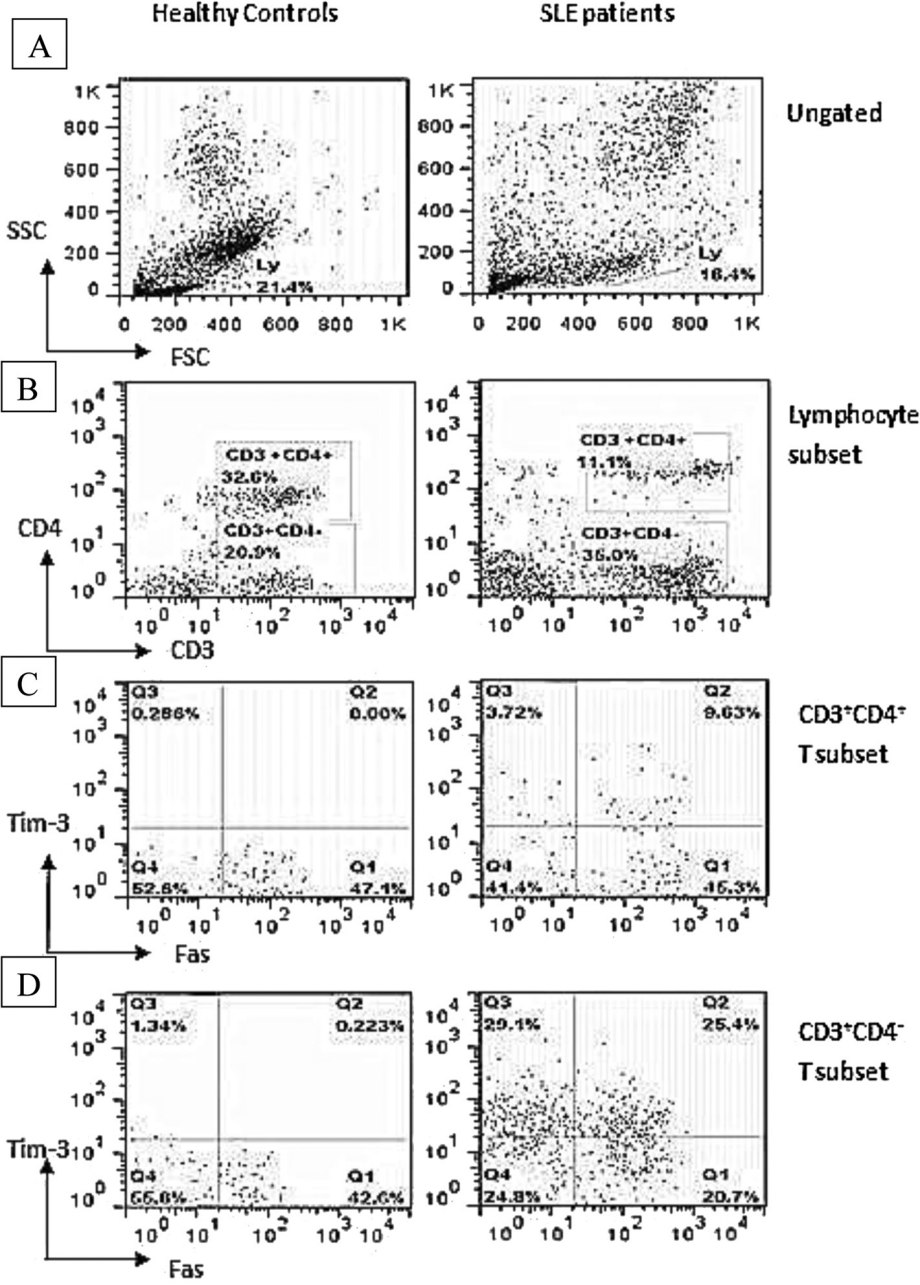
Fluorescence-activated cell sorting to quantify lymphocyte subsets from patients with SLE and healthy controls (HCs). Peripheral blood mononuclear cells from HC (left panels) and patients (right panels) were sorted to reveal proportions of (**a**) lymphocytes in the total cell population, (**b**) CD3^+^CD4^+^ and CD3^+^CD4^-^ T subsets among all lymphocytes, (**c**) Tim^+^ and Fas^+^ subsets among CD3^+^CD4^+^ lymphocytes and (**d**) Tim^+^ and Fas^+^ subsets among CD3^+^CD4^-^ lymphocytes

**Fig. 2:**
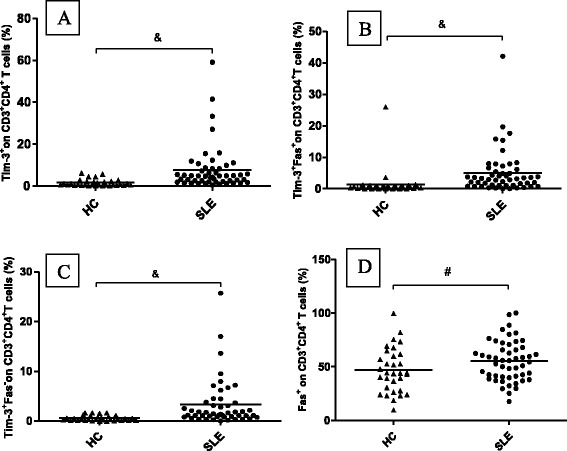
Tim-3 and Fas expression on CD3^+^CD4^+^ T cells in patients with SLE and healthy controls (HCs). CD3^+^CD4^+^ T cells from patients expressed significantly higher levels of (**a**) Tim-3^+^, (**b**) Tim-3^+^Fas^+^, and (**c**) Tim-3^+^Fas^-^. **d** Fas expression on CD3^+^CD4^+^ T cells was similar between patients and HCs. Horizontal bars indicate mean levels. &, *p* < 0.05; #, *p* > 0.05

**Fig. 3:**
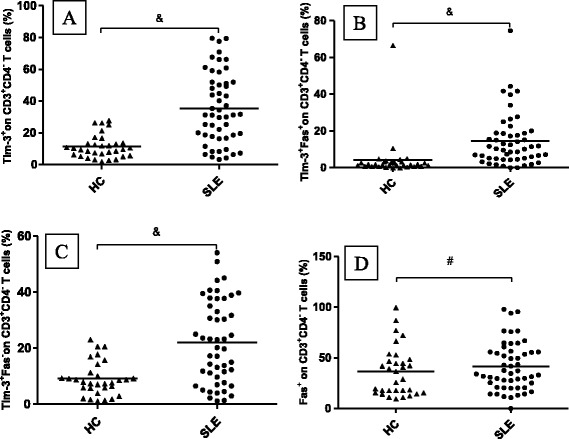
Tim-3 and Fas expression on CD3^+^CD4^-^T cells in patients with SLE and healthy controls (HCs). CD3^+^CD4^-^T cells from patients expressed significantly higher levels of (**a**) Tim-3^+^, (**b**) Tim-3^+^Fas^+^, and (**c**) Tim-3^+^Fas^-^. **d** Fas expression on CD3^+^CD4^-^ T cells was similar between patients and HCs. Horizontal bars indicate mean levels. &, *p* < 0.05; #, *p* > 0.05

Populations of lymphocytes, CD3^+^CD4^+^ T cells and CD3^+^CD4^-^ T cells in patients and HCs were gated using FACS (Fig. 1), and then the surface expression of Tim-3 and Fas on these subsets was assessed. Surface expression of Tim-3^+^, Tim-3^+^Fas^-^ and Tim-3^+^Fas^+^ was significantly higher on CD3^+^CD4^+^ T cells and CD3^+^CD4^-^ T cells in patients than on the corresponding cells in HCs (*p* < 0.001; Fig. 2a-c and Fig. 3a-c). In contrast, surface expression of Fas^+^ and Tim-3^-^Fas^+^ was similar on CD3^+^CD4^+^ T cells and CD3^+^CD4^-^ T cells in the two groups (Fig. 2d and Fig. 3d).

### Correlation of FasL and galectin-9 mRNA levels with Fas and Tim-3 expression in CD3^+^T cells

Expression of the Tim-3 ligand galectin-9 mRNA in PBMCs did not significantly correlate with Tim-3 surface expression on CD3^+^CD4^+^ or CD3^+^CD4^-^ T cells. Similar results were obtained for FasL mRNA levels and Fas expression (data not shown).

### Correlation between Tim-3 expression and SLE disease activity

Both SLEDAI and serum levels of complement proteins can reflect SLE disease activity, and SLEDAI score in our patients correlated negatively with serum levels of C_3_ (*r* = -0.448, *p* < 0.01) and C_4_ (*r* = -0.374, *p* < 0.05; Table 3). Serum levels of C_3_ and C_4_, in turn, correlated negatively with Tim-3 surface expression (*r* = -0.549, *p* < 0.01; *r* = -0.453, *p* < 0.01) and with Tim-3^+^Fas^+^ surface co-expression(*r* = -0.488, *p* < 0.01; *r* = -0.476, *p* < 0.01) on CD3^+^CD4^+^ T cells. Similarly, C_3_ and C_4_ levels negatively correlated with Tim-3^+^ expression (*r* = -0.513, *p* < 0.01; *r* = -0.416, *p* < 0.01) and Tim-3^+^Fas^+^ expression (*r* = -0.441, *p* < 0.01; *r* = -0.495, *p* < 0.01) on CD3^+^CD4^-^ T cells.

**Table 3:** Correlations between proportions of different T cell subsets and disease activity index in Chinese patients with SLE

	SLEDAI score	C_3_	C_4_
CD3^+^CD4^+^ T cells	-0.075	0.108	0.182
Fas^+^	0.213	0.076	0.022
Tim-3^+^Fas^+^	0.143	-0.488^b^	-0.476^b^
Tim-3^+^	0.178	-0.549^b^	-0.453^b^
CD3^+^CD4^-^ T cells	0.205	-0.125	0.124
Fas^+^	-0.025	0.084	-0.035
Tim-3^+^Fas^+^	0.112	-0.441^b^	-0.495^b^
Tim-3^+^	0.156	-0.513^b^	-0.416^b^
SLEDAI score	1.0	-0.448^b^	-0.374^a^
C_3_	-0.448^b^	1.0	0.716^b^
C_4_	-0.374^a^	0.716^b^	1.0

^a^Correlation significant at the 0.05 level (2-tailed)

^b^Correlation significant at the 0.01 level (2-tailed)

No correlation was found between surface Fas expression and SLEDAI score for any of the T subsets examined. Similarly, no correlation was found between FasL or Galectin-9 mRNA levels and SLEDAI score.

**Fig. 4:**
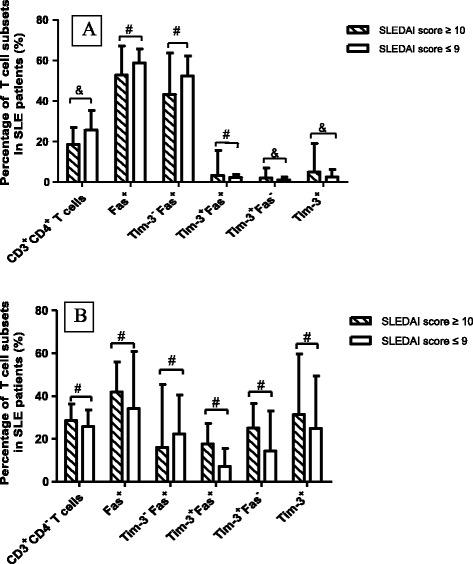
Comparison of proportions of different T cell subsets between patients with active or stable SLE. SLE patients were classified based on SLEDAI score as having active disease (score ≥ 10) or stable disease (score ≤ 9). **a** The CD3^+^CD4^+^ T subset made up a smaller proportion of lymphocytes in patients with active disease than in those with stable disease, while CD3^+^CD4^+^Tim-3^+^ and CD3^+^CD4^+^Tim-3^+^Fas^-^ subsets made up a larger proportion of lymphocytes in patients with active disease (&, *p* < 0.05). **b** Proportions of CD3^+^CD4^-^ T cells expressing Tim and Fas were similar between patients with active or stable SLE (#, *p* > 0.05)

Comparison of T cell subsets between patients with active or stable SLE showed that patients with active disease had significantly lower proportions of CD3^+^CD4^+^ T subsets and higher proportions of CD3^+^CD4^+^Tim-3^+^ and CD3^+^CD4^+^Tim-3^+^Fas^+^ cells (*p* = 0.01; Fig. 4).

## Discussion

In this case-control study, we compared the expression of Tim-3^+^, Tim-3^+^Fas^-^, Tim-3^+^Fas^+^, Tim-3^-^Fas^+^ and Fas^+^ on CD3^+^CD4^+^ T cells and CD3^+^CD4^-^T cells between patients with SLE and matched HCs. We found that expression of Tim-3^+^, Tim-3^+^Fas^-^ and Tim-3^+^Fas^+^ on CD3^+^CD4^+^ T cells and CD3^+^CD4^-^ T cells was significantly higher in patients. We did not, however, find significant correlations between FasL mRNA expression in PBMCs and Fas^+^ expression on CD3^+^CD4^+^orCD3^+^CD4^-^ T cells in patients; the same was observed for galectin-9 and Tim-3. We further found that SLEDAI score negatively correlated with serum levels of C_3_ and C_4_. These serum levels, in turn, correlated negatively with Tim-3^+^ and Tim-3^+^Fas^+^ expression on CD3^+^CD4^+^ and CD3^+^CD4^-^ T cells.

CD4^+^T cell subsets include Th1, Th2, Th17 and regulatory T cells (Treg), while CD8^+^ T cells are usually cytotoxic T cells. The CD3^+^CD4^-^ T cells in our study were mainly CD8^+^ T cells. Our study found a lower proportion of CD3^+^CD4^+^ T cells and higher proportion of CD3^+^CD4^-^ T cells in patients than in HCs, leading to an abnormal ratio of CD3^+^CD4^+^ T cells to CD3^+^CD4^-^ T cells. This is in accord with previous studies [28, 29], and it supports the observations that SLE involves an imbalance of Th1⁄Th2 [30] and that abnormal expression of key signaling molecules on T lymphocytes plays an important role in SLE pathogenesis [31, 32].

Indeed, abnormal T cell signaling is thought to contribute to SLE by altering gene transcription and cytokine production [31–33]. One of the affected pathways is apoptosis [5, 34]. Fas is an apoptosis-promoting cell surface receptor, and interactions between Fas and FasL play an essential role in maintaining immune homeostasis [35]. Fas expression on total peripheral blood lymphocytes has been reported to be higher in SLE patients than in HCs [11, 36, 37] and to correlate with both disease activity [11, 36]and organ damage [37]. Similarly, Amasaki et al. reported higher mean fluorescence intensity due to Fas expression on both CD4^+^ and CD8^+^ T cell subsets in patients than in HCs [38]. In the present study, however, Fas^+^Tim-3^+^ expression on CD3^+^CD4^+^ and CD3^+^CD4^-^ T cells was higher in patients than in HCs, but the difference was not significant. This discrepancy may reflect the difference between using percentage of fluorescent cells or mean fluorescence intensity as the outcome measure.

Increased Fas expression on lymphocytes because of lymphocyte activation may account for the increased susceptibility to Fas-mediated apoptosis in SLE. Induction of the T cell response can activate T cells or induce them to express Fas and FasL. Fas^+^ or FasL^+^ T lymphocytes can kill each other or commit suicide directly, and they can effect killing through autocrine and paracrine mechanisms [39]. Fas participates in removing reactive lymph cell clones and activated T cells from the circulation. Dysregulation or inhibition of Fas-mediated signaling leads to the accumulation of activated T cells, which can cause autoimmune diseases such as SLE.

While Fas/FasL is the main pathway for cell death in SLE pathogenesis, signaling by Tim-3/galectin-9 also contributes to lymphocyte apoptosis. Tim-3 is a powerful immunosuppressive molecule involved in immune tolerance and autoimmune responses. Tim-3 was initially identified on terminally differentiated Th1 cells and negatively regulates the T cell response by inducing T cell apoptosis [40]. Dysregulation of Tim-3 expression on CD4^+^ T cells and CD8^+^ T cells is closely related to autoimmune diseases [22, 41]. In the present study, Tim-3 expression on both CD3^+^CD4^+^ T cells and CD3^+^CD4^-^ T cells was significantly higher in patients than in HCs. In addition, Tim-3 expression on CD3^+^CD4^+^ T cells was higher in active SLE patients than in stable SLE ones. These findings contrast with previous work from our group showing no significant difference in Tim-3 expression on total PBMCs between patients and HCs [25]. This discrepancy may reflect the different expression of Tim-3 on various PBMC subsets other than CD3^+^CD4^+^and CD3^+^CD4^-^ T cells, including activated Th17 cells, macrophages/monocytes, dendritic cells, and natural killer cells. Future work should clarify what happens with Tim-3 expression in different PBMC subsets.

Our results show that Tim-3 expression on CD3^+^CD4^+^ and CD3^+^CD4^-^ T cells negatively correlated with serum levels of C_3_ and C_4_, which correlated in turn with SLEDAI score. As we know, C_3_ and C_4_ levels are SLE disease activity biomarkers. Thus, the positive correlations between C_3_ or C_4_ and Tim-3 expression on T cells, especially the CD3^+^CD4^+^ T subset suggest that Tim-3 expression maybe can reflect SLE disease activity as well as C_3_ and C_4_ levels. Since increased Tim-3 expression can negatively regulate Th1 cell responses and decrease inflammation-induced organ damage, the higher expression in patients with SLE may reflect in part a compensatory response to the inflammation in SLE. Future studies should examine whether the observed higher Tim-3 expression in patients with SLE helps drive SLE pathogenesis, compensate for SLE-related inflammation, or both.

FasL belongs to the tumor necrosis factor ligand family and induces pro-apoptotic signals by binding to Fas [10]. Inhibition of Fas/FasL-mediated apoptosis by circulating anti-FasL autoantibody may contribute to the immune abnormalities and pathogenesis of SLE [42]. Over-expression of membrane-bound Fas ligand (CD95L) exacerbated autoimmune disease and renal pathology in pristane-induced lupus [43], and administering anti-FasL monoclonal antibody to an NZB/W F1 mouse model of lupus prevented the development of lupus nephritis [44]. Our data did not show a significant correlation between FasL mRNA expression and proportions of CD3^+^CD4^+^ Fas^+^ or CD3^+^CD4^-^ Fas^+^ T cells. This discrepancy may reflect the fact that Fas is abundantly expressed in various cell types.

The Tim-3 ligand, galectin-9, has attracted attention for playing various roles in innate and adaptive immunity [45]. The Tim-3/galectin-9 pathway regulates Th1 immunity, resulting in apoptosis of Th1 cells [21, 46]. Treating mouse models of experimental autoimmune arthritis with galectin-9 ameliorated disease [17]. In contrast, our data showed no correlation between galectin-9 mRNA expression and proportions of CD3^+^CD4^+^Tim-3^+^ or CD3^+^CD4^-^Tim-3^+^ T cell subsets. This discrepancy may reflect Tim-3-independent activity of galectin-9 under our experimental conditions. Galectin-9 can also bind CD40 [46] and other receptors on T-cells [47], including protein disulfide isomerase [48]. In addition, galectin-9 suppresses Th17 cell development via a mechanism that is independent of Tim-3 but dependent on IL-2. Again independently of Tim-3, galectin-9 has been shown to ameliorate clinical severity of lupus-prone MRL/lpr mice by inducing plasma cell apoptosis [49]. In fact, one study found evidence that Tim-3 does not act as a galectin-9 receptor [50]. Future studies should confirm that galectin-9 functions via Tim-3-dependent and-independent mechanisms.

While our data on expression of Tim-3 and Fas are consistent with the idea that T cells play an important role in SLE by amplifying the autoimmune response [51], we caution that we examined only CD3^+^CD4^+^ and CD3^+^CD4^-^ T cell subsets. Several other T cell subsets have been implicated in SLE pathogenesis, including Th17 cells [52, 53], Treg cells [54–56], T follicular helper (TFH) cells [57, 58], and DN T cells [59, 60]. In addition, we did not measure proportions of apoptotic cells or whether Tim-3 is expressed at higher levels in those cells than in non-apoptotic ones. Tim-3 and Fas are known to be co-expressed in some T cell types, such as Treg and Th17. Future studies should examine Tim-3 and Fas expression in these and other T subsets to gain a clearer picture of their potential usefulness as a biomarker of SLE activity.

## Conclusions

In conclusion, our data indicate that patients with SLE contain higher proportions of CD3^+^CD4^+^Tim-3^+^ and CD3^+^CD4^-^Tim-3^+^ T cell subsets than HCs, and Tim-3 expression on CD3^+^CD4^+^ and CD3^+^CD4^-^ T cells correlates with SLE disease activity. Our data suggest that Tim-3 and Fas can be co-expressed on the same T cell subsets, and that the proportion of Tim^+^Fas^+^ cells is higher in active SLE than stable SLE. This suggests a close association between Tim-3 expression and T cell apoptosis in patients with active SLE, and highlights the need for future research to clarify how this association contributes to disease pathogenesis.
